# Efficacy and safety of TCMs with anti-inflammatory effect in patients with rheumatoid arthritis: A network meta-analysis

**DOI:** 10.3389/fimmu.2023.1114930

**Published:** 2023-03-08

**Authors:** Jinying Fang, Mingxuan Liu, Zhenghui Huang, Yucao Ma, Yiwen Wang, Xiaojia Zheng, Liu Lv, Chunpin Liu, Wei Li, Zhenghong Zhu, Huachao Zhu, Jie Hu, Yonghong Wang, Hailong Wang

**Affiliations:** Department of Rheumatology, Dongzhimen Hospital, Beijing University of Chinese Medicine, Beijing, China

**Keywords:** traditional Chinese medicines, Tripterygium wilfordii Hook F, Sinomenine, rheumatoid arthritis, network meta-analysis

## Abstract

**Background:**

Traditional Chinese medicines (TCMs), such as Tripterygium wilfordii Hook F (TwHF), Glycyrrhiza uralensis, Caulis sinomenii and others have anti-inflammatory effects. They are widely used in China to treat rheumatoid arthritis (RA), but proof of their use as an evidence-based medicine is little. The aim of this network meta-analysis (NMA) was to evaluate the efficacy and safety of TCMs.

**Methods:**

By searching online databases and using a manual retrieval method, randomized controlled trials (RCTs) that met specific selection criteria were included in the meta-analysis. The search included papers that were published between the establishment of the databases and November 10, 2022. Analyses were performed using Stata software (version 14) and Review Manager (version 5.3).

**Results:**

61 papers with 6316 subjects were included in the current NMA. For ACR20, MTX plus SIN therapy (94.30%) may be a significant choice. For ACR50 and ACR70, MTX plus IGU therapy (95.10%, 75.90% respectively) performed better than other therapies. IGU plus SIN therapy (94.80%) may be the most promising way to reduce DAS-28, followed by MTX plus IGU therapy (92.80%) and TwHF plus IGU therapy (83.80%). In the analysis of the incidence of adverse events, MTX plus XF therapy (92.50%) had the least potential, while LEF therapy (22.10%) may cause more adverse events. At the same time, TwHF therapy, KX therapy, XF therapy and ZQFTN therapy were not inferior to MTX therapy.

**Conclusions:**

TCMs with anti-inflammatory effect were not inferior to MTX therapy in the treatment of RA patients. Combining with TCMs can improve the clinic efficacy and reduce the possibility of adverse events of DMARDs, which may be a promising regimen.

**Systematic review registration:**

https://www.crd.york.ac.uk/PROSPERO/, identifier CRD42022313569.

## 1 Introduction

Rheumatoid arthritis (RA) is a common chronic inflammatory disease that leads to severe joint damage, disability and low quality of life. What’s more, RA patients may suffer from serious comorbidities, such as lung disease, cardiovascular disease, osteoporosis, etc. RA is a worldwide social and economic burden, as it occurs in approximately 5-10 per 1000 people (1–3). Many studies have shown disease-modifying anti-rheumatic drugs (DMARDs) could prevent or reduce joint damage, and maintain normal joint function. Although, various types of DMARDs have been used for RA patients, including conventional synthetic DMARDs, targeted synthetic DMARDs and biologic DMARDs, more than 40% of patients could not control the progression of RA or tolerate adverse effects after taking DMARDs (4, 5). Therefore, more and more attention has been paid to traditional Chinese medicines (TCMs), which have a long history in the treatment of RA. TCMs, such as Tripterygium wilfordii Hook F (TwHF), Glycyrrhiza uralensis, and Caulis sinomenii, have anti-inflammatory and anti-angiogenic effects (6–9).

TwHF preparation is widely used in RA patients in China. A project (2017YFC0907604) from The Chinese National Key Research R&D Program found that among 82,589 RA patients, 16.5% were taking TwHF, ranking fourth just behind methotrexate (MTX), leflunomide (LEF) and hydroxychloroquine at the end of November 10, 2022. The recent randomized control trial (RCT) reported by Yang-Zhong Zhou et al. (10) illustrates TwHF has similar efficacy in the treatment of RA compared to MTX in the American College of Rheumatology 20% (ACR20), ACR50, ACR70 assessment. Triptolide is the main bioactive component of TwHF and is primarily responsible for the anti-inflammatory effect. Some breakthroughs in molecular biology are related to triptolide, which helps us in using TwHF preparation (11, 12).

Kunxian capsule and Xinfeng capsule are the new generations of TwHF preparations, which could relieve joint pain, joint swelling and morning stiffness. They could reduce toxicity and increase efficacy by adding matrimony vine, Astragalus membranaceus, Epimedium, centipede, etc (13, 14). Sinomenine (SIN) and its new preparation, Zhengqing Fengtongning capsule, also have an anti-inflammatory function to inhibit the progression of RA (9). However, large-scale RCTs are still lacking, especially data on the efficacy and safety of various TCMs compared with other DMARDs.

This network meta-analysis (NMA) differs from a traditional meta-analysis in that it may directly or indirectly compare the efficacy of multiple interventions simultaneously. This NMA aims to compare the efficacy and safety of various TCMs with DMARDs in RA patients. The results will provide a basis for strengthening conclusions to guide and support the clinical use of the TCMs with anti-inflammatory effects in RA patients.

## 2 Methods

### 2.1 Inclusion criteria

#### 2.1.1 Study design

RCTs investigating the treatment of RA with anti-inflammatory TCMs, published in either English or Chinese, regardless of the use of the blind method. The study protocol was registered with PROSPERO (CRD42022313569).

#### 2.1.2 Participants

Participants were defined as having been diagnosed with RA according to the 1987 American Rheumatology Association guidelines (15) or the 2010 ACR/European League against Rheumatism (EULAR) criteria (16).

#### 2.1.3 Interventions

Tripterygium Glycoside Tablet or Tripterygium Tablet (TwHF), Kunxian Capsule (KX), Xinfeng Capsule (XF), Zhengqing Fengtongning Capsule (ZQFTN), SIN, MTX, LEF, Iguratimod (IGU), Sulfasalazine (SSZ) used singly or as a two-drug combination in the treatment of RA. The duration of treatment was not less than 8 weeks. There is to be no limitation on the use of non-steroidal anti-inflammatory drugs, folic acid, calcium tablets, vitamins, and low-dose hormones during the treatment.

#### 2.1.4 Outcome measures

ACR20, ACR50, ACR70, disease activity score in 28 joints (DAS-28) and adverse event.

### 2.2 Exclusion criteria

(1) patients with other autoimmune diseases or other serious conditions that could influence the results, such as severe heart failure, cancer, DIC, and severe infections; (2) studies that were abstracts, case reports, reviews, commentaries, and editorials, etc.; (3) literature with repetitive content; or (4) interventions such as herbs containing.

### 2.3 Data sources and searches

This study used PubMed, Web of Science, the Cochrane Library, Excerpta Medica Database (EMBASE), VIP Information/Chinese Scientific Journals, China Network Knowledge Infrastructure (CNKI), and WANFANG databases to search for relevant studies. The literature search included articles that were published between the establishment of the databases and November 10, 2022.

We conducted electronic searches using exploded Medical Subject Headings (MeSH) terms and various combinations of the keywords. The search terms used were (MeSH exp “Rheumatoid arthritis” and key words “Caplan Syndrome”, “Felty Syndrome”, “Rheumatoid Nodule”, “Rheumatoid Vasculitis”, “Sjogren’s Syndrome”, “Rheumatic Diseases”, “Rheumatic”, and “Rheumatic Diseases*”), (MeSH exp “tripterygium” and key words “Tripterygium* wilfordii”, “Tripterygium* wilfordius”, “Leigong Teng*”, “Leigong Teng*”, and “Thundergod Vine*”), “Kunxian Capsule”, “Xinfeng Capsule”, “Zhengqing Fengtongning Capsule”, “SIN”, “Methotrexate”, “Leflunomide”, “Iguratimod”, “Sulfasalazine”. At the same time, reference lists of included textbooks, all retrieved studies, review articles, and reports of academic congresses were checked manually. A comprehensive search strategy is shown in **Supplementary Table S3**. For the Chinese databases, free texts were used, such as “Lei gong teng”, “Lei Gong TengZhiji”, “Lei Gong Teng Duo Gan”, “Jia An Die Ling (MTX)”,”Lai Fu Mi Te (LEF)”, “Kun Xian Jiao Nang (Kunxian Capsule)”, “Xin Feng Jiao Nang (Xinfeng Capsule)”, “Zheng Qing Feng Tong Ning (Zhengqing Fengtongning Capsule)”, “Ai La Mo De (Leflunomide)”, “Liu Dan Huang An Pi Ding (Sulfasalazine)”, “Qing Teng Jian (Sinomenine)”, “Sui Ji Dui Zhao Shi Yan (RCT)”, “Lei feng shi guan jie yan (rheumatoid arthritis)”.

### 2.4 Study extractions and quality assessment

Two investigators (Fang and Liu) independently reviewed the studies from the retrieved literature, based on the inclusion criteria, and extracted their analytical results and data. If two investigators had different opinions about the quality of a study, a third author (Huang) reassessed the differences. Data were only included for review only when a consensus was reached among all three investigators.

Two investigators (Fang and Liu) independently assessed the risk of bias using the Cochrane risk-of-bias tool. Each study was reviewed and scored as high (if the answer was yes), low (if the answer was no), or unclear (if there were insufficient details to make a definite judgment), based on the following criteria: (1) selection bias (random sequence generation), (2) selection bias (random sequence generation), (3) performance bias (blinding of participants and personnel), (4) detection bias (blinding of outcome assessment), (5) attrition bias (incomplete outcome data), (6) reporting bias (selective reporting), and (7) other bias.

### 2.5 Data synthesis and analysis

The endpoints of this NMA were ACR20, ACR50, ACR70, DAS-28 and adverse events. ACR20 is defined as a reduction by 20% or greater reduction in the number of tender and swollen joints plus a 20% improvement in at least three of the following five measures: pain, patient global assessment, physician global assessment, physical disability score, and blood acute-phase reactants. ACR50 is defined as an reduction of 50% or greater reduction in the number of tender and swollen joints, plus 50% improvement in at least three of the aforementioned five measures. ACR70 is defined as an reduction of 70% or greater reduction in the number of tender and swollen joints, plus 70% improvement in at least three of the above five measures. Disease Activity Score in 28 joints (DAS-28) is related to tender and swollen joints, Erythrocyte Sedimentation Rate (ESR) and general health (GH). The formula is as following:


DAS−28= 0.56∗sqrt (tender joints 28)        + 0.28∗sqrt (swollen joints 28)+0.70∗Ln (ESR)+0.014∗GH

### 2.6 Statistical analysis

In this meta-analysis, the mean differences (MD) were chosen as the effect sizes for continuous outcomes. The weighted mean difference ((WMD = √SD1^2^ plus SD2^2^ – SD1 × SD2); SD = baseline endpoint) was used to evaluate measured data. For dichotomous outcomes, pooled odds ratios (OR) were calculated with corresponding 95% confidence intervals (CI). Because of the clinical and methodological heterogeneity of both treatments and subjects among those enrolled trials, random-effect models were used in the meta-analysis (17, 18). First, the Bayesian NMA was performed using STATA software (version 14). The network graph could display the relationship among different interventions for each outcome. For example, line thicknesses indicated the number of trials and the node sizes corresponded to the total sample sizes for treatments. The hierarchy of treatment rankings was estimated by the value of surface under the cumulative ranking curve (SUCRA), in which the more enormous SUCRA value of comparisons was regarded as the better efficacy or the lower the adverse event (19). Using the loop-specific approach evaluates the agreement between direct and indirect sources of evidence in each closed loop (20). Publication bias was assessed using funnel plots and consistency tests.

## 3 Results

### 3.1 Background information on the included studies

**Figure 1:**
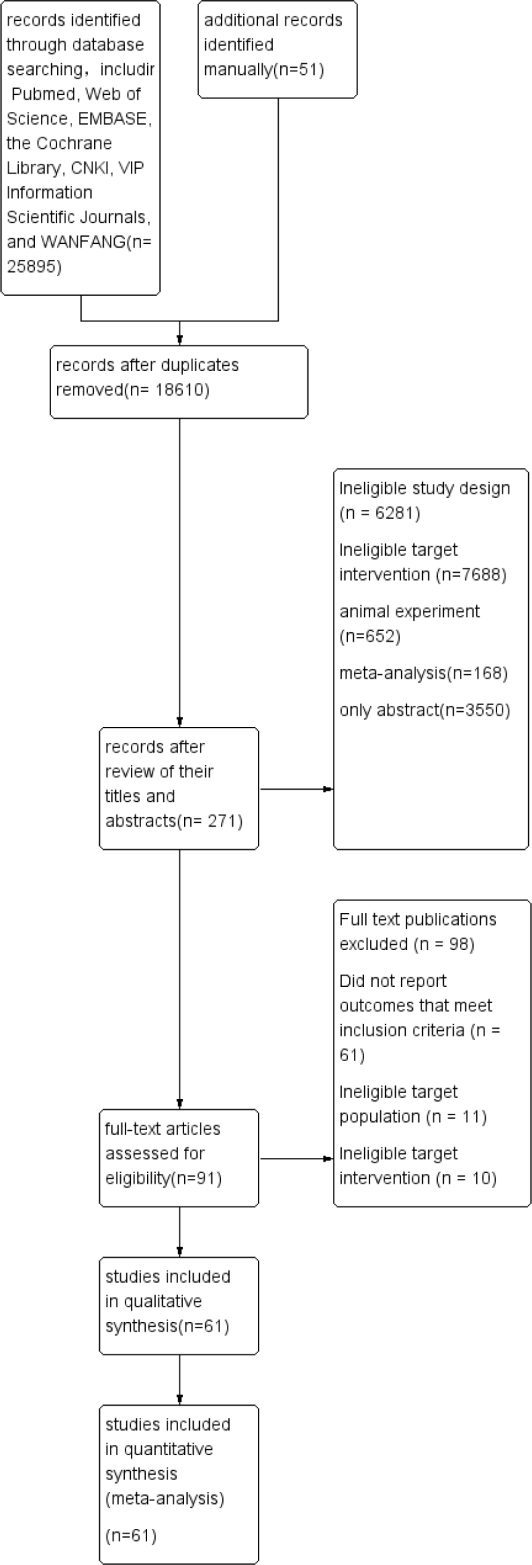
Flow diagram of study identification, inclusion, and exclusion.

**Figure 2:**
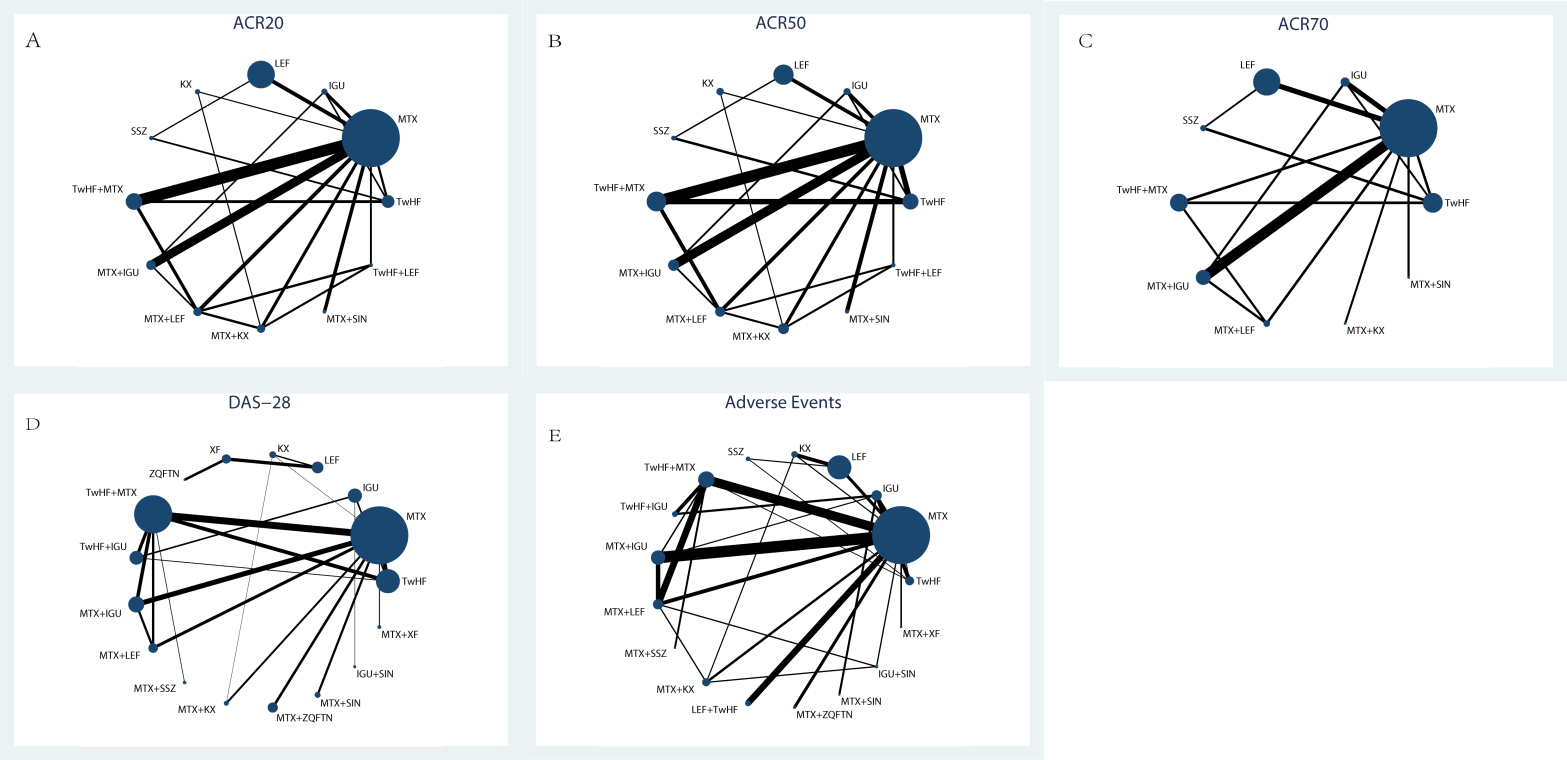
The evidence network of all papers about different treatments. **(A)** ACR20; **(B)** ACR50; **(C)** ACR70; **(D)** DAS-28; **(E)** Adverse Events. Line thicknesses corresponded to the number of trials, and node sizes indicated the total sample sizes for treatments.

Electronic and manual searches yielded 25895 potentially relevant papers; 2613 from Pubmed, 1657 from Web of Science,1786 from the EMBASE, 1416 from the Cochrane Library, 7213 from CNKI, 5980 from VIP Information/Chinese Scientific Journals, 5179 from WANFANG, and 51 manually. After removing duplicated papers, 18610 papers remained. Of these, 271 papers were selected after reviewing their titles and abstracts. After reviewing the full text of each publication and based on the exclusion criteria, 91 papers were selected. After reviewing the studies included in the qualitative synthesis, 61 papers were selected, including 6316 patients (10, 14, 21–79). The selection process is shown in **Figure 1**. Detailed information on the included studies is provided in **Supplementary Table S1**. The entire network plots of different comparisons for all outcomes are shown in **Figure 2**.

### 3.2 Risk of bias

**Figure 3:**
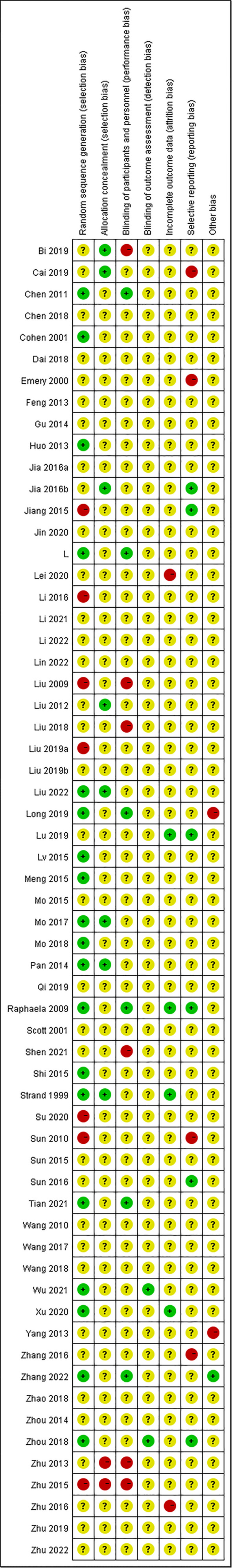
Risk of bias graph: each risk of bias item for each included study.

The overall quality of the studies included in this review was un satisfactory; details of the risk-of-bias assessment are shown in **Figure 3**. All 61 papers used the random number acquisition method, but none offered a detailed description. Of the 61 papers included, 20 used random number table, 1 used the computer random method and 1 used bicolor random.

### 3.3 NMA results

#### 3.3.1 ACR20

**Figure 4:**
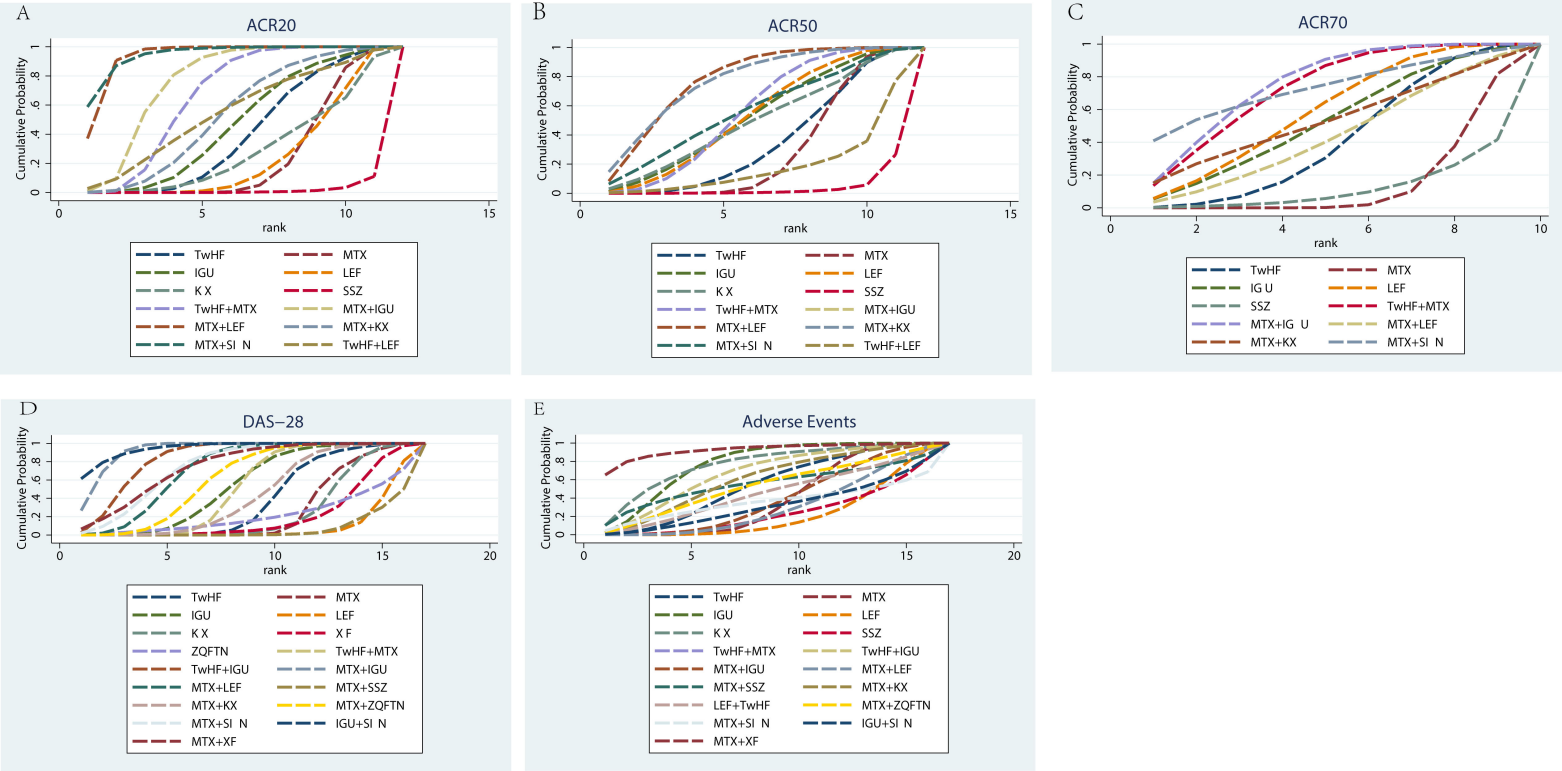
Rank of the cumulative probabilities for basic parameters. **(A)** ACR20; **(B)** ACR50; **(C)** ACR70; **(D)** DAS-28; **(E)** Adverse Events.

In the evaluation of the ACR20, 28 studies were included with a total of 3630 patients. There were 12 types of interventions, including TwHF therapy, MTX therapy, IGU therapy, KX therapy, LEF therapy, SSZ therapy, MTX plus TwHF therapy, MTX plus KX therapy, MTX plus LEF therapy, TwHF plus LEF therapy, MTX plus IGU therapy and MTX plus SIN therapy, as shown in **Figure 4A**. MTX plus SIN therapy (94.30%) exhibited the most significant possibility of improving ACR20 over other treatments, followed by MTX plus LEF therapy (93.30%) and MTX plus IGU therapy (76.00%). For single drug therapy, KX therapy (28.20%) and TwHF therapy (39.20%) were not inferior to MTX therapy (23.80%) and LEF therapy (23.60%) in improving ACR20, as shown in **Table 1**.

**Table 1:** The SUCRA values of each treatment.

Treatment	ACR20	Rank	ACR50	Rank	ACR70	Rank	DAS-28	Rank	Adverse Events	Rank
TwHF	39.20%	8	34.60%	9	41.50%	8	38.10%	11	54.60%	7
MTX	23.80%	10	29.10%	10	14.70%	9	26.00%	12	39.60%	11
IGU	46.40%	7	52.10%	7	53.00%	6	53.80%	8	77.10%	2
LEF	23.60%	11	53.00%	6	59.50%	4	9.10%	17	22.10%	17
KX	28.20%	9	49%	8	—	—	24.60%	13	76.80%	3
SSZ	1.60%	12	3.40%	12	11.70%	10	—	—	24.60%	16
XF	—	—	—	—	—	—	20.20%	15	—	—
ZQFTN	—	—	—	—	—	—	21.50%	14	—	—
TwHF+MTX	66.30%	4	55.40%	5	73.00%	3	51.50%	9	40.80%	10
TwHF+IGU	—	—	—	—	—	—	83.80%	3	67.20%	4
MTX+IGU	76.00%	3	95.10%	1	75.90%	1	92.80%	2	39.20%	12
MTX+LEF	93.30%	2	77.30%	2	44.10%	7	71.50%	6	31.10%	15
MTX+SSZ	—	—	—	—	—	—	6.90%	16	55.60%	6
MTX+KX	53.20%	6	76.40%	3	53.40%	5	43.70%	10	60.80%	5
MTX+ZQFTN	—	—	—	—	—	—	61.60%	7	53.60%	8
MTX+SIN	94.30%	1	56.20%	4	73.10%	2	75.00%	5	36.10%	13
IGU+SIN	—	—	—	—	—	—	94.80%	1	32.50%	14
TwHF+LEF	54.20%	5	18.20%	11	—	—	—	—	45.70%	9
MTX+XF	—	—	—	—	—	—	75.10%	4	92.50%	1

#### 3.3.2 ACR50

Regarding the ACR50, 27 studies were included with a total of 3032 patients. There were 12 types of interventions, including TwHF therapy, MTX therapy, IGU therapy, KX therapy, LEF therapy, SSZ therapy, MTX plus TwHF therapy, MTX plus KX therapy, MTX plus LEF therapy, TwHF plus LEF therapy, MTX plus IGU therapy and MTX plus SIN therapy, as shown in **Figure 4B**. MTX plus IGU therapy (95.10%), MTX plus LEF therapy (77.30%) and MTX plus KX therapy (76.40%) did better in improving ACR50 than other therapies, as shown in **Table 1**. For single drug therapy, TwHF therapy (34.60%) was not inferior to MTX therapy (29.10%) in improving ACR50. Meanwhile, KX therapy (49.00%) and LEF therapy (53.00%) were similar in ACR50.

#### 3.3.3 ACR70

In the evaluation of the ACR70, 19 studies were included with a total of 2348 patients. There were 10 types of interventions, including TwHF therapy, MTX therapy, IGU therapy, LEF therapy, SSZ therapy, MTX plus TwHF therapy, MTX plus KX therapy, MTX plus LEF therapy, MTX plus IGU therapy and MTX plus SIN therapy, as shown in **Figure 4C**. MTX plus IGU therapy (75.90%), MTX plus SIN therapy (73.10%) and TwHF plus MTX therapy (73.00%) did better in improving ACR70 than other therapies, as shown in **Table 1**.

#### 3.3.4 DAS-28

In the evaluation of the DAS-28, 40 studies were included with a total of 3418 patients. There were 17 types of interventions, including TwHF therapy, MTX therapy, IGU therapy, KX therapy, LEF therapy, ZQFTN therapy, XF therapy, MTX plus TwHF therapy, MTX plus KX therapy, MTX plus LEF therapy, TwHF plus IGU therapy, MTX plus ZQFTN therapy, MTX plus IGU therapy, MTX plus SSZ therapy, MTX plus SIN therapy, IGU plus SIN therapy, and MTX plus XF therapy, as shown in **Figure 4D**. IGU plus SIN therapy (94.80%) had the most significant possibility of improving DAS-28 over other treatments, followed by MTX plus IGU therapy (92.80%) and TwHF plus IGU therapy (83.80%). For single drug therapy, TwHF therapy (38.10%), KX therapy (24.60%), XF therapy (20.20%) and ZQFTN therapy (21.50%) were not inferior to MTX therapy (26.00%) in improving DAS-28, as shown in **Table 1**.

#### 3.3.5 Adverse events

In the incidence of adverse events, 47 studies were included with a total of 5111 patients, including gastrointestinal reactions, liver dysfunction, allergic reactions, headache, etc, as shown in **Supplementary Table S2**. There were 17 types of interventions, including TwHF therapy, MTX therapy, IGU therapy, KX therapy, LEF therapy, SSZ therapy, MTX plus TwHF therapy, MTX plus KX therapy, MTX plus LEF therapy, TwHF plus IGU therapy, MTX plus ZQFTN therapy, MTX plus IGU therapy, MTX plus SSZ therapy, TwHF plus LEF therapy, MTX plus SIN therapy, IGU plus SIN therapy, and MTX plus XF therapy (**Figure 4E**). The SUCRA values of each treatment for adverse events showed MTX plus XF therapy (92.50%) had the least potential for the incidence of adverse events than other treatments, while LEF therapy (22.10%) may cause more adverse events, as shown in **Table 1**.

#### 3.3.6 Forest plots

In this study, a forest plot was generated to assess for inconsistency, as shown in **Supplementary Figure S1**. With the exception of the M-S-T closed loop with all outcomes, there was no apparent inconsistency in any of the other closed loops, as shown in **Table 2**.

**Table 2:** the closed loop with all outcomes.

Outcomes	Loop	*P* (CI)
ACR20	MTX-TwHF-TwHF+MTX	0.218(0.00,2.31)
	MTX-MTX+KX-TwHF+LEF	0.559(0.00,2.44)
	MTX-MTX+KX-MTX+LEF	0.615(0.00,2.74)
	KX-MTX-MTX+KX	0.478(0.00,2.11)
	MTX-MTX+LEF-TwHF+MTX	0.965(0.00,1.94)
ACR50	TwHF-MTX-TwHF+MTX	0.196(0.00,2.46)
	MTX-MTX+KX-TwHF+LEF	0.769(0.00,1.91)
	MTX-MTX+LEF-MTX+KX	0.767(0.00,1.90)
	MTX-KX-MTX+KX	0.720(0.00,1.61)
	MTX-SSZ-MTX+LEF	0.992(0.00,2.68)
DAS-28	IGU-TwHF-TwHF+IGU	0.534(0.00,0.85)
	MTX-TwHF-TwHF+MTX	0.773(0.00,1.19)
	KX-MTX-MTX+KX	0.731(0.00,1.01)
	TwHF-TwHF+IGU-TwHF+MTX	0.883(0.00,1.51)
Adverse Events	MTX-MTX+KX-MTX+LEF	0.075(0.00,2.87)
	IGU+SIN-MTX-MTX+LEF	0.123(0.00,3.10)
	LEF-MTX-SSZ-TwHF	0.168(0.00,2.92)
	IGU-MTX+IGU-TwHF+IGU-TwHF+MTX	0.338(0.00,2.99)
	KX-LEF-MTX	0.569(0.00,4.30)
	MTX-MTX+LEF-TwHF+MTX	0.350(0.00,2.66)
	IGU-MTX+IGU-TwHF-TwHF+MTX	0.476(0.00,2.62)
	MTX-MTX+IGU-TwHF+MTX	0.467(0.00,2.23)
	MTX-TwHF-TwHF+MTX	0.689(0.00,2.65)
	IGU-MTX-TwHF	0.627(0.00,1.67)
	IGU-TwHF-TwHF+IGU-TwHF+MTX	0.746(0.00,1.98)
	MTX+IGU-MTX+LEF-TwHF+MTX	0.856(0.00,3.08)
	IGU-MTX-TwHF+IGU-TwHF+MTX	0.772(0.00,1.88)
	IGU-MTX-MTX+IGU	0.780(0.00,1.43)
	MTX-MTX+IGU-MTX+LEF	0.994(0.00,1.76)
	IGU+SIN-MTX-MTX+KX	1.000(0.00,1.80)
	KX-MTX-MTX+KX	1.000(0.00,1.73)

#### 3.3.7 Cluster analysis

**Figure 5:**
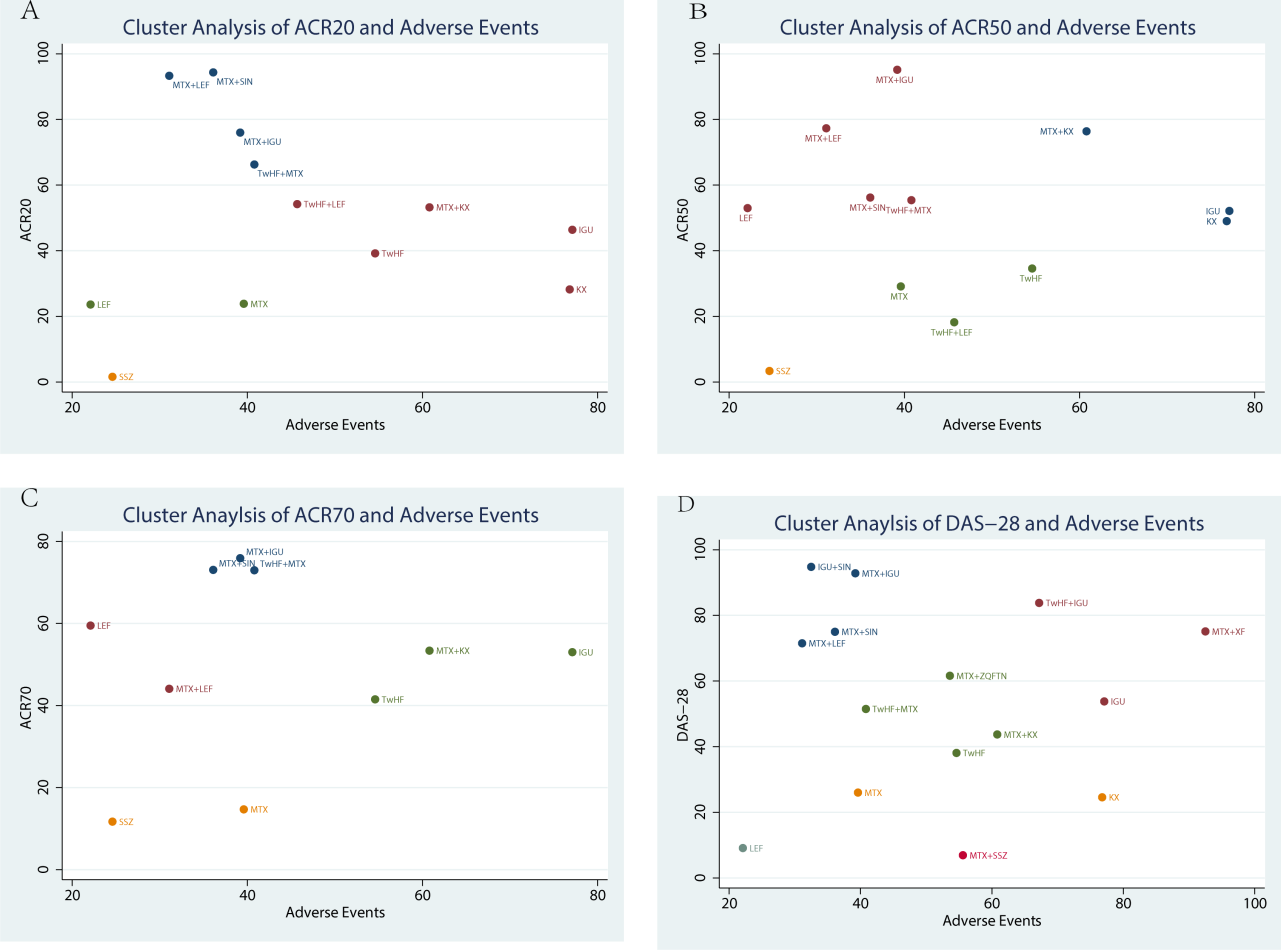
Cluster analysis plot of efficacy and safety. **(A)** the adverse events (X-axis) and ACR20 (Y-axis); **(B)** the adverse events (X-axis) and ACR50 (Y-axis); **(C)** the adverse events (X-axis) and ACR70(Y-axis); **(D)** the adverse events (X-axis) and DAS-28 (Y-axis).

Cluster analysis was used to identify the most promising therapeutic strategies among the different treatments in terms of ACR20, ACR50, ACR70, DAS-28 and the incidence of adverse events simultaneously. As shown in **Figure 5A**, the results of the cluster analysis showed that MTX plus KX therapy and IGU therapy were associated with favorable benefits both for improving the ACR20 and reducing the incidence of adverse events compared with the other treatments. In contrast, LEF therapy and SSZ therapy were the worst treatments in improving ACR20 and reducing the incidence of adverse events, as shown in **Figure 5A**. MTX plus KX therapy had the greatest possibility of improving ACR50 and reducing adverse events, while SSZ therapy was the worst treatment, as shown in **Figure 5B**. For improving ACR70 and reducing the adverse events, IGU therapy was the most effective treatment, while SSZ therapy was the worst treatment, as shown in **Figure 5C**. For DAS-28 and adverse events, MTX plus XF therapy was the most effective treatment, while LEF therapy was the worst treatments, as shown in **Figure 5D**.

#### 3.3.8 Publication bias

**Figure 6:**
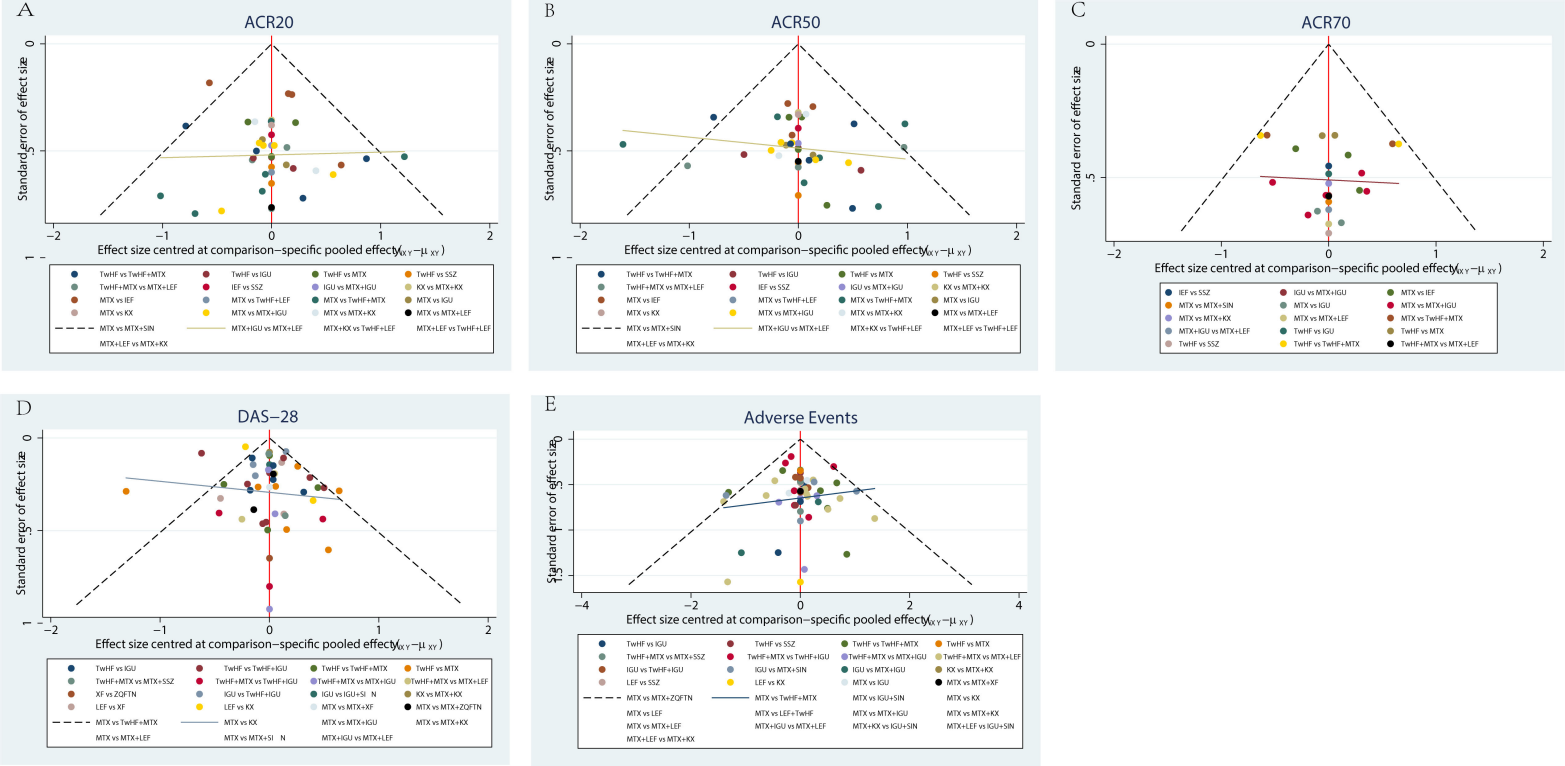
Funnel plot. **(A)** ACR20; **(B)** ACR50; **(C)** ACR70; **(D)** DAS-28; **(E)** Adverse Events.

According to the funnel plot of ACR20, ACR50, ACR70, DAS-28 and adverse events, this NMA may have a potential publication bias in the present study, as shown in **Figure 6**.

## 4 Discussion

There was no consensus on the use of TCMs in combination with other DMARDs in the treatment of RA. This NMA was conducted to generate a hierarchy of treatment rankings. The ranking probabilities for these treatments were further calculated in terms of their clinical efficacy and safety under in various endpoints to provide a basis for making better optimal choices. This NMA found that for ACR20, MTX plus SIN therapy (94.30%) had the greatest likelihood of achieving the best efficacy among the treatment regimens involved, followed by MTX plus LEF therapy (93.30%) and MTX plus IGU therapy (76.00%). For ACR50 response, MTX plus IGU therapy (95.10%), MTX plus LEF therapy (77.30%), and MTX plus KX therapy (76.40%) performed better than other therapies. For ACR70, MTX plus IGU therapy (75.90%) ranked first. In the assessment of DAS-28, IGU plus SIN therapy (94.80%) had the greatest potential to improve DAS-28 over other treatments, followed by MTX plus IGU therapy (92.80%) and TwHF plus IGU therapy (83.80%). In the analysis of the incidence of adverse events, MTX plus XF therapy (92.50%) had the least potential, while LEF therapy (22.10%) may cause more adverse events. At the same time, TwHF therapy, KX therapy, XF therapy and ZQFTN therapy were not inferior to MTX therapy. According to the results of cluster analysis, MTX plus KX therapy and MTX plus XF therapy may be good choices in terms of both efficacy and safety. We found that combining with TCMs could improve the clinical efficacy and reduce the possibility of adverse events of other DMARDs.

RA is a chronic inflammatory autoimmune disease, but why it occurs is still uncertain as yet. In general, we believe that occurrence of RA is related to genetic, epigenetic and environmental factors (80). However, the most important factor is the inflammatory immune response, which is characterized by an increase in inflammatory cytokines and chemokines, circulating autoantibodies and increasing concentration (81). Synovial hyperplasia is the main pathological manifestation of RA, which is the main cause of joint damage (81). A network pharmacology found the mechanisms of TwHF would treat RA through 31 signaling pathways, and then it inactivates TNF and NF-kappa B signaling pathways to inhibit the inflammatory response (82). RA-synovial fibroblasts (RASFs) are the main factors of joint destruction in RA (83). The main component of Kunxian Capsule and Xinfeng Capsule is triptolide, which is extracted from TwHF. TwHF inhibited PGE2 production by IL-1β-stimulated RASFs and inhibited COX-2 protein expression. At the same time, it can inhibit lipopolysaccharide-induced chemokine CCL5 production by RASFs and induce apoptosis of RASFs by activating caspase-3 activity (84–86). TwHF reduces the production of IL-1, IL-17, and TNF-α, which would protect chondrocytes (87). Also, Tripterygium wilfordii significantly inhibits the transcription and generation of IL-17 from murine splenocytes and purified CD4+ T cells by repressing IL-6-induced phosphorylation of STAT3 (88). The animal experiments also show that it could apparently relieve joint pain and swelling by suppressing the releases of IL-1α, IL-1βIL-4, IL-10 and MMP3 (89).

Sinomenine (SIN) is extracted from CAULIS SINOMENII, which is the main active ingredient of Zhengqing Fengtongning capsule (90). Many studies demonstrated sinomenine could alleviate morning stiffness and joint pain in the RA patients, by modifying neurotransmission, inhibiting cyclooxygenase 2-dependent prostaglandin E2 and NO, TNF, INF-γ, IL-6, IL-1β, and IL-4, and suppressing the activity of P38 MAPK, MMPs, and NF-κB (91, 92). At the same time, SIN could regulate immunity and alleviate inflammation by inhibiting T- lymphocyte and B-lymphocyte activation (93), regulating NLRP3 inflammasome and NF-κB pathway (94). Our NMA showed, Zhengqing Fengtongning capsule combined with MTX could obviously reduce the DAS-28 of RA patients. Thus, Zhengqing Fengtongning capsule may be a good choice for RA patients, who want to reduce morning stiffness and joint pain.

This NMA had some limitations. First, during the literature review, we found that not all studies described the allocation concealment procedure or the randomization process in great detail; so selection bias could not be completely excluded. Second, the number of articles with different treatment methods varies widely, which may lead to relatively imprecise conclusions. In the same time, the sample size of some studies was small and the treatment cycle was short. Next, some treatments had few papers, which may lead to statistical bias. Finally, TCMs are not widely used in other countries. Therefore, almost all selected papers in this NMA were from China, which may have caused regional, language, and racial biases. We hope that in the future there will be large-scale RCTs in different countries to further provide more reliable data.

## 5 Conclusion

According to the results of ACR20, MTX plus SIN therapy may be the significant choice for RA patients. According to the results of ACR50 and ACR70, MTX plus IGU therapy may be the significant choice for RA patients. According to the results of DAS-28, IGU plus SIN therapy had the greatest possibility. According to the results of safety, MTX plus XF therapy had the least potential for the incidence of adverse events. MTX plus KX therapy and MTX plus XF therapy may be good choices, considering both efficacy and safety. In conclusion, combining with TCMs can improve the clinical efficacy and reduce the possibility of adverse events of DMARDs, which may be a promising regimen.

## Data availability statement

The original contributions presented in the study are included in the article/**Supplementary Material**, further inquiries can be directed to the corresponding author/s.

## Author contributions

The article was designed by H-LW. J-YF drafted the manuscript and data curation with support of H-LW. M-XL and Z-HH contributed to data curation, validation, and writing review. All authors contributed to the scientific discussion of the integrative approach and of the manuscript. All authors contributed to the article and approved the submitted version.
