# Supplementary material for: Efficacy and safety of TCMs with anti-inflammatory effect in patients with rheumatoid arthritis: A network meta-analysis
Source: Front Immunol. 2023 Mar 8;14:1114930. doi: 10.3389/fimmu.2023.1114930 (PMC10030962; doi:10.3389/fimmu.2023.1114930)
Supplement: Supplementary file 1 [file DataSheet_1.docx]

Supplementary Material

**Efficacy and safety of TCMs with anti-inflammatory effect in patients with rheumatoid arthritis: a network meta-analysis**

**Jin-ying Fang, Ming-xuan Liu, Zheng-hui Huang, Yu-cao Ma, Yi-wen Wang, Xiao-jia Zheng, Liu Lv, Chun-pin Liu, Wei Li, Zheng-hong Zhu, Chao-hua Zhu, Jie Hu, Yong-hong Wang, Hai-long Wang^*^**


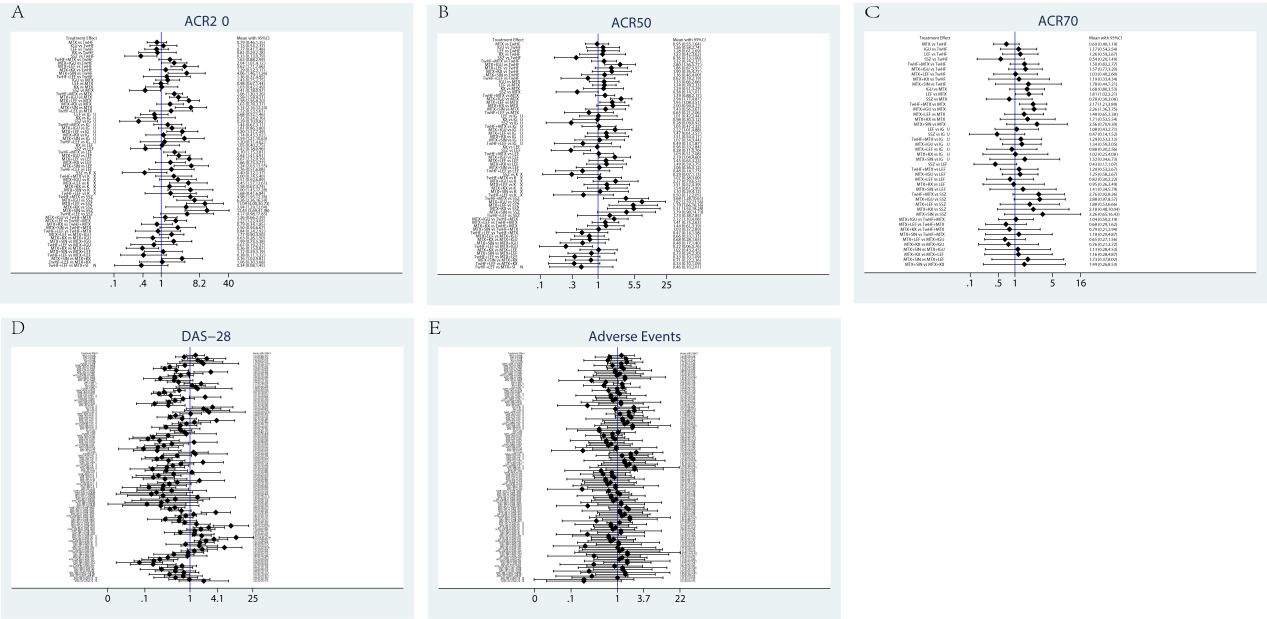


Supplementary Figure S1. Forest Plots. Note: A: ACR20; B: ACR50; C: ACR70; D:DAS-28; E: adverse events.

| Supplementary Table S1 Basic information of all the papers | | | | | | | | | | | | | | |
| --- | --- | --- | --- | --- | --- | --- | --- | --- | --- | --- | --- | --- | --- | --- |
|  |  |  | Intervention | | | Gender (M/F) | | | Average age(years) | | |  |  |  |
| Paper | Sample size (Control group/Treatment group/Other groups) | Gender (M/F) | Control group | Treatment group | Other groups | Control group | Treatment group | Other groups | Control group | Treatment group | Other groups | Duration of treatment（week） | Outcomes | randomization method |
| Feng 2013^[21]^ | 20/22 | 4/38 | MTX | MTX+TwHF | — | 2/18 | 2/18 | — | 52 ± 3.8 | 51 ± 4.2 | — | 12 | ①⑤ | random |
| Sun 2010^[22]^ | 40/40 | — | MTX+LEF | MTX+TwHF | — | — | — | — | — | — | — | 24 | ①②⑤ | random |
| Tian 2021^[23]^ | 59/61 | 39/81 | MTX | MTX+TwHF | — | 19/40 | 20/41 | — | 68.07 ± 4.45 | 68.12 ± 4.47 | — | 24 | ④ | random number table |
| Zhu 2015^[24]^ | 29/41 | 23/47 | MTX | MTX+TwHF | — | 9/20 | 14/27 | — | 46.81 ± 16.05 | 47.68 ± 15.33 | — | 8 | ④ | random |
| Yang 2013^[25]^ | 40/40 | 10/70 | MTX | MTX+TwHF | — | 4/36 | 6/34 | — | 68.79 ± 7.44 | 69.32 ± 6.93 | — | 24 | ①②⑤ | random |
| Jia 2016^[26]^ | 30/30 | 17/43 | MTX | MTX+KX | — | 8/22 | 9/21 | — | 20~70 | 22~70 | — | 12 | ①②③④⑤ | random |
| Jiang 2015^[27]^ | 28/28 | — | LEF | KX | — | — | — | — | — | — | — | 12 | ④⑤ | random |
| Xu 2020^[28]^ | 20/20/20 | 12/48 | MTX | KX | MTX+KX | — | — | — | — | — | — | 24 | ④ | random number table |
| Jiang 2018^[29]^ | 26/26 | 14/38 | TwHF+LEF | MTX+LEF | — | 6/20 | 8/18 | — | 54.4 ± 0.6 | 53.2 ± 0.8 | — | 24 | ①②⑤ | random |
| Liu 2019b^[30]^ | 30/50 | 6/74 | LEF | KX | — | 2/28 | 4/46 | — | 35~59 | 36~62 | — | 12 | ④ | random |
| Zhu 2016^[31]^ | 30/30 | — | MTX+TwHF | MTX+LEF | — | — | — | — | — | — | — | 8 | ①②③④⑤ | random |
| Chen 2011^[32]^ | 34/34 | 20/48 | MTX+LEF | MTX+TwHF | — | 11/23 | 9/25 | — | 41.2 ± 15.8 | 36.9 ± 16.0 | — | 12 | ④⑤ | random |
| Pan 2014^[33]^ | 20/20 | 5/35 | MTX | MTX+TwHF | — | 3/17 | 2/18 | — | 41.3 ± 5.1 | 45.8 ± 6.3 | — | 12 | ①②⑤ | random number table |
| Long 2019^[34]^ | 30/30 | 14/46 | MTX | MTX+TwHF | — | 6/24 | 8/22 | — | 64.79 ± 4.51 | 65.03 ± 4.74 | — | 12 | ①②⑤ | random number table |
| Wang 2018^[35]^ | 15/14/15 | — | MTX | TwHF | MTX+TwHF | — | — | — | 53.7 ± 14.2 | 54.1 ± 14 | 55.5 ± 13 | 12 | ②④ | random |
| Lei 2020^[36]^ | 32/32 | 20/44 | MTX | MTX+TwHF | — | 9/23 | 11/21 | — | 66.1 ± 5.9 | 64.5 ± 6.3 | — | 24 | ④⑤ | random |
| Wang 2010^[37]^ | 120/66 | 66/120 | MTX | ZQFTN+MTX | — | 23/43 | 43/77 | — | 42.5 | 41.2 | — | 24 | ④⑤ | random |
| Lin 2022^[38]^ | 80/79/79 | 56/182 | KX | MTX | MTX+KX | 24/56 | 13/66 | 19/60 | 52.14 ± 9.7 | 48.62 ± 13.01 | 51.76 ± 11.67 | 12 | ①②⑤ | random |
| Liu 2009^[39]^ | 20/20 | 14/26 | ZQFTN | XF | — | 8/12 | 6/14 | — | 43.1 ± 11.92 | 40.35 ± 13.24 | — | 4 | ④ | random |
| Sun 2016^[40]^ | 50/50 | 6/94 | LEF | XF | — | 5/45 | 1/49 | — | 38.26 ± 11.24 | 40.32 ± 9.35 | — | 12 | ④ | random number table |
| Sun 2015^[41]^ | 50/50 | 6/94 | LEF | XF | — | — | — | — | — | — | — | 12 | ④ | random |
| Zhang 2016^[14]^ | 30/30 | 20/40 | LEF | XF | — | 10/25 | 5/25 | — | — | — | — | 12 | ④ | random |
| Zhu 2013^[42]^ | 36/36 | 21/51 | MTX | MTX+ZQFTN | — | — | — | — | — | — | — | 24 | ④⑤ | random |
| Lu 2019^[43]^ | 40/40 | 19/61 | MTX | MTX+ZQFTN | — | 11/29 | 8/32 | — | 47.13 ± 14.46 | 46.38 ± 13.53 | — | 12 | ④⑤ | random |
| Liu 2018^[44]^ | 45/45 | 30/60 | TwHF | TwHF+IGU | — | 14/31 | 16/29 | — | 65.2 ± 4.51 | 66.64 ± 4.17 | — | 24 | ④ | random |
| Wu 2021^[45]^ | 54/54 | — | IGU | TwHF+IGU | — | — | — | — | 44.8 ± 6.6 | 45.2 ± 6.4 | — | 36 | ④⑤ | random number table |
| Li 2016^[46]^ | 40/44 | 34/50 | MTX+TwHF | MTX+IGU | — | 16/24 | 18/26 | — | 60～82 | 60～77 | — | 12 | ④⑤ | random |
| Liu 2019a ^[47]^ | 36/36 | 14/58 | TwHF | IGU | — | 8/28 | 8/28 | — | — | — | — | 12 | ①②③④⑤ | random |
| Su 2020^[48]^ | 40/40 | — | TwHF | IGU | — | — | — | — | 51.1 ± 10.2 | 50.88 ± 11.4 | — | 24 | ④⑤ | random |
| Li 2021^[49]^ | 30/30 | 19/41 | MTX+TwHF | MTX+IGU | — | 11/19 | 8/22 | — | 47.56 ± 8.13 | 45.32 ± 7.44 | — | 24 | ④⑤ | random |
| Mo 2018^[50]^ | 30/30 | 14/46 | MTX+TwHF | MTX+IGU | — | 6/24 | 8/22 | — | 43.3 ± 10.25 | 45 ± 11.56 | — | 12 | ④⑤ | random number table |
| Shen 2021^[51]^ | 50/50 | 38/62 | IGU | TwHF+IGU | — | 20/30 | 18/32 | — | 64. 75 ± 5. 64 | 65. 17 ± 6. 02 | — | 12 | ④⑤ | random |
| Zhou 2018^[10]^ | 69/69/69 | — | MTX | TwHF | MTX+TwHF | — | — | — | — | — | — | 24 | ①②③④ | random |
| Raphaela 2009^[52]^ | 60/61 | 23/98 | TwHF | SSZ | — | 16/44 | 7/54 | — | 54 ± 11 | 52 ± 12 | — | 24 | ①②③⑤ | random number table |
| Fan 2018^[53]^ | 45/45 | 43/47 | MTX+SSZ | MTX+TwHF | — | 21/24 | 22/23 | — | 52.89±9.92 | 53.32±10.21 | — | 16 | ④⑤ | random |
| Zhou 2014 ^[54]^ | 30/30 | 23/37 | MTX | MTX+SIN | — | 11/19 | 12/18 | — | 41.3 | 42.5 | — | 8 | ④ | random |
| Dai 2018^[55]^ | 60/60 | 71/49 | MTX | MTX+SIN | — | 35/25 | 36/24 | — | 51.17±4.6 | 52.39±4.57 | — | 12 | ④ | random number table |
| Gu 2014^[56]^ | 45/45 | 23/67 | MTX | MTX+SIN | — | 12/33 | 11/34 | — | 39.84 ± 10.81 | 40.95 ± 9.40 | — | 24 | ①②⑤ | random number table |
| Cai 2019^[57]^ | 29/30 | 14/45 | MTX | MTX+SIN | — | 7/22 | 7/23 | — | 55±13 | 53.0±10 | — | 12 | ①②③⑤ | random number table |
| Zhang 2022^[58]^ | 37/37 | 35/39 | IGU | IGU+SIN | — | 6/31 | 8/29 | — | 47.5±3.21 | 37.46±3.17 | — | 12 | ④⑤ | random number table |
| Lv 2015^[59]^ | 69/69/69 | 37/170 | MTX | TwHF | MTX+TwHF | 10/59 | 13/56 | 14/55 | 51 | 51.3 | 50.6 | 24 | ①②③④⑤ | computer random method |
| Wang 2017^[60]^ | 60/60 | 31/89 | MTX | MTX+IGU | — | 17/43 | 14/46 | — | 41. 37 ± 2. 16 | 40. 86 ± 2. 45 | — | 16 | ④⑤ | random number table |
| Mo 2015^[61]^ | 30/30 | 17/43 | MTX | MTX+IGU | — | 9/21 | 8/22 | — | 31.9 ± 8.6 | 31.8 ± 8.5 | — | 12 | ①②③⑤ | random number table |
| Li 2022^[62]^ | 40/40 | 19/61 | MTX | MTX+IGU | — | 10/30 | 9/31 | — | 49.96 ± 5.71 | 49.94 ± 5.73 | — | 24 | ①②③⑤ | random number table |
| Zhao 2018^[63]^ | 36/36 | 25/47 | MTX | MTX+IGU | — | 13/23 | 12/24 | — | 46.90 ± 3.60 | 47.20 ± 3.40 | — | 12 | ④⑤ | bicolor random |
| Zhu 2019^[64]^ | 30/30 | 8/52 | MTX | IGU | — | 3/27 | 5/25 | — | — | — | — | 24 | ①②③⑤ | random |
| Zhu 2022^[65]^ | 51/51 | 14/88 | MTX | MTX+XF | — | 8/43 | 6/45 | — | 56.75 ± 8.46 | 57.02 ± 9.28 | — | 12 | ④⑤ | random number table |
| Jin 2020^[66]^ | 46/46 | 41/51 | MTX | MTX+IGU | — | 21/25 | 20/26 | — | 49.87 ± 9.78 | 50.03 ± 9.96 | — | 24 | ④⑤ | random number table |
| Meng 2015^[67]^ | 33/33 | 11/55 | MTX+LEF | MTX+IGU | — | 7/26 | 4/29 | — | 41. 7 ± 22. 8 | 44. 2 ± 20. 5 | — | 16 | ①②③④⑤ | random number table |
| Shi 2015^[68]^ | 30/30 | 18/42 | MTX | MTX+IGU | — | 10/20 | 8/22 | — | 48.4 ± 10.2 | 48.9 ± 12.2 | — | 24 | ①②③④⑤ | random number table |
| Chen 2018^[69]^ | 40/40 | 24/56 | MTX | MTX+IGU | — | — | — | — | — | — | — | 8 | ①②③⑤ | random number table |
| Mo 2017^[70]^ | 30/30 | 19/41 | MTX | MTX+LEF | — | 9/21 | 10/20 | — | 52. 5 ± 3. 4 | 53. 3 ± 3. 6 | — | 12 | ④⑤ | random |
| Qi 2019^[71]^ | 40/40/40 | — | MTX | IGU | MTX+IGU | — | — | — | — | — | — | 24 | ①②③⑤ | random |
| Liu 2012^[72]^ | 48/48 | 44/52 | LEF | MTX | — | — | — | — | — | — | — | 24 | ①②③⑤ | random |
| Huo 2013^[73]^ | 38/38 | 22/54 | MTX | MTX+LEF |  | 12/26 | 10/28 | — | 49 ± 11 | 48 ± 12 | — | 24 | ①②③⑤ | random |
| Bi 2019^[74]^ | 30/30 | 12/48 | MTX+LEF | MTX+IGU | — | 6/24 | 6/24 | — | 54.6 ± 11.88 | 53.10 ± 12.9 | — | 12 | ④⑤ | random |
| Scott 2001^[75]^ | 60/60 | — | SSZ | LEF | — | — | — | — | — | — | — | 96 | ①②③⑤ | random |
| Emery 2000^[76]^ | 286/314 | — | LEF | MTX | — | — | — | — | — | — | — | 96 | ①⑤ | random |
| Strand 1999 ^[77]^ | 182/182 | 95/269 | LEF | MTX | — | 50/132 | 45/137 | — | — | — | — | 52 | 1. ③⑤ | random |
| Cohen 2001^[78]^ | 190/190 | — | LEF | MTX | — | 51/139 | 49/141 | — | 54 | 53 | — | 96 | 1. ③⑤ | random |
| Liu 2022^[79]^ | 27/27 | 23/31 | IGU | TwHF+IGU | — | 11/16 | 12/15 | — | 50.14 ± 3.77 | 49.95 ± 3.78 | — | 12 | ④ | random |
| Note: MTX: Methotrexate; LEF: Leflunomide; TwHF: tripterygium glycoside tablet or tripterygium tablet; XF:Xinfeng capsule; KX: Kunxian capsule; IGU:Iguratimod; SSZ:Sulfasalazine; SIN:Sinomenine; ZQFTN: Zhengqing Fengtongning capsule; XF: Xinfeng capsule. ①: ACR20; ②: ACR50; ③: ACR70; ④: DAS-28; ⑤:Adverse Events. | | | | | | | | | | | | | | |
|  |  |  |  |  |  |  |  |  |  |  |  |  |  |  |

| Table S2. adverse events of different treatments* | | | | | | | | | | | | | | | | | | |
| --- | --- | --- | --- | --- | --- | --- | --- | --- | --- | --- | --- | --- | --- | --- | --- | --- | --- | --- |
| treatment | Hepatic dysfunction (n) | Local swelling and painresponder (n) | Reinfection (n) | Gastrointestinal reaction (n) | Rash (n) | Oral ulcer (n) | Leucopenia (n) | Menstrual disturbance (n) | Insomnia (n) | Alopecia (n) | Headache, dizziness (n) | Fatigue(n) | Allergy (n) | Anemia (n) | Thrombocytopenia (n) | Hypertension (n) | total adverse events (n)* | Total (n) |
| IGU | 15 | — | — | 22 | 5 | — | 5 | 4 | 0 | 0 | 5 | — | 1 | — | — | — | 59 | 287 |
| TwHF+IGU | 6 | — | — | 12 | — | — | 0 | 5 | 0 | 0 | 2 | — | — | — | — | — | 25 | 104 |
| TwHF+MTX | 17 | — | 1 | 17 | 5 | 3 | 12 | 1 | 0 | 2 | 9 | 2 | — | — | — | — | 118 | 462 |
| MTX+IGU | 21 | — | — | 35 | 5 | 1 | 16 | — | 1 | — | 6 | — | — | — | — | — | 86 | 489 |
| TwHF | 5 | — | — | 6 | 1 | — | 1 | 4 | — | — | — | — | — | — | — | — | 80 | 205 |
| MTX | 48 | — | 4 | 64 | 13 | 3 | 27 | 1 | — | 3 | 5 | — | 1 | 1 | 2 | — | 375 | 1741 |
| MTX+LEF | 23 | — | — | 25 | 5 | 2 | 6 | — | — | 2 | 3 | 2 | — | — | — | — | 68 | 261 |
| MTX+KX | 5 | — | 1 | 8 | — | — | 1 | 2 | — | 2 | 2 | — | — | — | — | — | 21 | 109 |
| LEF | 1 | — | — | 5 | 1 | — | 1 | — | — | 5 | 1 | — | — | — | — | 3 | 169 | 794 |
| KX | — | — | — | 7 | — | — | — | 1 | — | — | 1 | — | — | — | — | — | 9 | 108 |
| TwHF+LEF | 2 | — | — | 4 | 2 | — | 2 | — | — | 1 | — | — | — | — | — | — | 11 | 26 |
| SSZ^*^ | — | — | — | 11 | 2 | — | — | — | — | — | — | — | — | — | — | 2 | 52 | 121 |
| ZQFTN+MTX | 1 | — | — | 2 | 10 | — | — | — | — | — | — | — | 1 | — | — | — | 14 | 196 |
| MTX+SIN | 1 | 4 | 1 | 2 | 1 | — | 2 | — | — | — | — | — | — | — | — | — | 11 | 75 |
| IGU+SIN | — | — | — | 2 | — | — | 1 | — | — | — | — | — | 1 | — | — | — | 4 | 37 |
| MTX+XF | 1 | — | — | 1 | — | — | — | — | — | — | — | — | — | — | 1 | — | 3 | 51 |
| MTX+SSZ | 1 | — | — | 2 | — | — | 1 | — | — | — | — | — | — | — | — | — | 4 | 45 |
| *Some papers did not report the specific types of adverse events and some reported multiple adverse events in one patient. | | | | | | | | | | | | | | | | | | |

Supplementary Table S3. PubMed search strategy

| Databases: | PubMed |  |  |  |  |  |  |  | |
| --- | --- | --- | --- | --- | --- | --- | --- | --- | --- |
| Search number | Search query | | | | | | | | Search fields |
| #1 | ((((((((((( Rheumatoid Arthritis )) OR ( Rheumatoid arthrosis ) ) OR ( arthrosis deformans)) OR ( Arthritis deformans )) OR ( beauvais disease)) OR ( Rheumatic Arthritis ) ) OR ( Rheumatic arthrosis ) ) OR ( Rheumatoid polyarthritis ) ) OR (rheumarthritis)) OR (caplan's syndrome)) OR (felty's syndrome)) | | | | | | | | All fields |
| #2 | ((((((((((((Randomized controlled trial) OR Randomized controlled trials) OR random allocation) OR random allocate) OR randomly allocate) OR double-blind method) OR single-blind method) OR double blind) OR single blind) OR triple blind) OR clinical trial) OR clinical trials) NOT animal | | | | | | | | All fields |
| #3 | ((((((((((((((((((((((((tripterygium)) OR (tripterygium hypoglaucum)) OR (tripterygiumhypoglaucums ) ) OR ( tripterygium wilfordii ) ) OR ( tripterygium wilfordius )) OR wilfordius ) ) OR ( Leigong Teng )) OR ( Lei gong Tengs )) OR ( lei gong teng ) ) OR ( leigongteng ) ) OR (Thundergod Vine)) OR (Thundergod Vines)) OR ( thunder god vine)) OR ( thundergodvine)) OR ( Tripterygium)) OR ( Triptolid)) OR (triptolide)) OR (tripterin)) OR (Tripdiolide)) OR (Tripdiolid)) OR (Triptonide)) OR (wilfordine)) OR (tripterin))) | | | | | | | | All fields |
| #4 | (Xinfeng capsule) OR (Xinfeng capsule) OR (Xin feng capsule) OR (xin feng capsule) OR ((Xin feng) OR ((Xinfeng) OR ((Xinfeng) OR ((xinfeng) | | | | | | | | All fields |
| #5 | (Zheng Qing Feng Tong Ning capsule) OR (Zheng Qing Feng Tong Ning) OR Zhengqing Fengtongning capsule) OR (Zhengqing Fengtongning) | | | | | | | | All fields |
| #6 | total glucosides of paeony OR TGP OR paeony | | | | | | | | All fields |
| #7 | Kun Xian Jiao Nang OR Kunxian capsule OR Kun xian capsule OR Kunxian OR Kun xian OR KX capsule OR KX | | | | | | | | All fields |
| #8 | #1 AND #2 AND (#3 OR #4 OR #5 OR #6 OR #7) | | | | | | | | All fields |
| #9 | Azathioprine OR Imurek OR Imurel OR AZA | | | | | | | | All fields |
| #10 | Cyclophosphamide OR Endoxan OR Cytoxan OR Neosar OR Procytox OR Revimmune OR CTX | | | | | | | | All fields |
| #11 | mycophenolate mofetil OR mycophenolate OR CellCept OR MMF | | | | | | | | All fields |
| #12 | tacrolimus OR fujimycin OR Prograf OR Advagraf OR Protopic OR FK506 | | | | | | | | All fields |
| #13 | intramuscular gold | | | | | | | | All fields |
| #14 | Auranofin OR Aureotan OR Auromyose OR Aktil OR Crisofin OR Crisinar OR Ridauran OR Ridaura OR Solganal OR SKF39162 | | | | | | | | All fields |
| #15 | Minocycline OR Klinomycin OR Mincmycin OR Minocyn OR Minomax OR Minomyc | | | | | | | | All fields |
| #16 | D-penicillamin | | | | | | | | All fields |
| #17 | Chlorambucil OR Leukeran OR Amboclorin OR Chloraminophene OR Chlorbutin OR CB-1348 OR NSC-3088 | | | | | | | | All fields |
| #18 | (#9 OR #10 OR #11 OR #12 OR #13 OR #14 OR #15 OR #16 OR #17 OR #18) AND #1 AND #2 | | | | | | | | All fields |
| #19 | #8 OR #18 | | | | | | | | All fields |
